# PLGA Nanoparticles Loaded with Sorafenib Combined with Thermosensitive Hydrogel System and Microwave Hyperthermia for Multiple Sensitized Radiotherapy

**DOI:** 10.3390/pharmaceutics15020487

**Published:** 2023-02-01

**Authors:** Ziqi Wang, Bo Liu, Jingyao Tu, Jingfeng Xiang, Hui Xiong, Yue Wu, Shuaijie Ding, Daoming Zhu, Dongyong Zhu, Fei Liu, Guangyuan Hu, Xianglin Yuan

**Affiliations:** 1 Department of Oncology, Tongji Hospital, Tongji Medical College, Huazhong University of Science and Technology, Wuhan 430030, China; 2 Guangdong Provincial Key Laboratory of Precision Medicine for Gastrointestinal Tumor and Department of General Surgery, Nanfang Hospital, The First School of Clinical Medicine, Southern Medical University, Guangzhou 510515, China; 3 Department of Thoracic Surgery, Tongji Hospital, Tongji Medical College, Huazhong University of Science and Technology, Wuhan 430030, China; 4 Department of Urology, Tongji Hospital, Tongji Medical College, Huazhong University of Science and Technology, Wuhan 430030, China; 5 Department of Gastrointestinal Surgery and Department of Geriatrics, Shenzhen People’s Hospital (The Second Clinical Medical College, Jinan University, The First Affiliated Hospital, Southern University of Science and Technology), Shenzhen 518020, China; 6 Department of Radiology, Union Hospital, Tongji Medical College, Huazhong University of Science and Technology, Wuhan 430030, China

**Keywords:** sorafenib, PLGA nanoparticles, microwave hyperthermia, radiotherapy, breast cancer

## Abstract

Hypoxia is typically the leading cause of radiotherapy (RT) resistance in solid tumors, and glutathione (GSH) overexpression in tumor cells is a potent antioxidant mechanism that protects tumor cells from radiation damage. Herein, we developed a sorafenib (SFN) loaded-PLGA hydrogel system (SPH) in combination with microwave (MW) hyperthermia for RT sensitization. SPH with stable properties was produced by combining SFN and PLGA in a specific ratio and encapsulating the mixture in agarose hydrogel. Intratumoral injection of SPH to mice combined with MW hyperthermia can not only directly cause thermal damage to tumor cells, but also increase blood oxygen delivery to the tumor site, thus overcoming the problem of intratumoral hypoxia and achieving “first layer” RT sensitization. Moreover, high temperatures can cause the hydrogel to disintegrate and release SFN. Not only can SFN inhibit tumor growth, but it can also achieve the “second layer” of RT sensitization by inhibiting glutathione (GSH) synthesis in cells and increasing reactive oxygen species (ROS) production. Experiments, both in vitro and in vivo, have indicated that SPH and MW hyperthermia can achieve a double RT sensitization effect and a significant tumor inhibition effect. In conclusion, combining our SPH nanosystem and thermoradiotherapy is a promising anti-tumor treatment.

## 1. Introduction

Breast cancer is the most prevalent female malignant tumor worldwide, accounting for 7–10% of all systemic malignant tumors, and is notorious for its low survival rate and high metastasis rate [1,2,3,4]. Cancer’s main clinical treatment methods include surgical excision, radiotherapy (RT), and chemotherapy [5,6]. RT is among the most prevalent and efficient cancer treatments. Utilizing high-energy X-rays or gamma rays induces radiation-induced DNA damage, produces reactive oxygen species (ROS), induces apoptosis, and then destroys the tumor by activating MAPK and P53 channels [7]. Due to the low radiation absorption capacity of tumor tissues, however, the use of high-energy radiation in the treatment process is likely to harm normal tissues [8]. To address this issue, lower radiation doses are frequently combined with radiation sensitizers to improve the efficacy of the treatment. Although numerous strategies have been devoted to developing effective RT sensitizers, the tumor microenvironment still shows inevitable changes in radiation sensitivity [9]. According to studies, the radiosensitivity of cells under aerobic conditions is approximately 2.5–3 times that of cells under hypoxia, meaning that a greater radiation dose is required to kill hypoxic cells [10,11]. In addition, additional factors may influence the effect of RT. For instance, glutathione overexpression facilitates the removal of reactive oxygen cells (ROS) generated by ionic radiation, thereby reducing the lethal effect of RT [12,13]. Furthermore, the sensitivity of cells with different division cycles to radiation is very different, with the radiation resistance of G1/S phase cells being thrice that of G2/M [14,15]. Consequently, developing effective RT sensitizers combined with various therapeutic means may enhance the radiation sensitivity of tumor cells, thereby enhancing the efficacy of tumor RT.

As the fifth therapy after surgery, radiation therapy, chemotherapy, and immunotherapy, hyperthermia is gaining importance in the comprehensive treatment of tumors [16]. Photothermal therapy (PTT) based on photosensitizers can convert near-infrared light energy into heat, causing environmental overheating and inducing apoptosis in cancer cells. Nevertheless, poor light penetration severely restricts the efficacy of PTT, not only for deep tumors, but also for larger superficial tumors [17,18,19]. Microwave (MW) therapy uses MW (a type of electromagnetic spectrum much lower than infrared light) energy, which is absorbed by tumor tissues with high water content and dielectric constant. It produces a thermal effect that causes the tumor tissues to rise to the effective therapeutic temperature and maintain it for some time, killing cancer cells without harming normal cells [20,21,22]. Due to its deep tissue penetration, high heating efficiency, and few side effects, MW hyperthermia is widely used in clinical tumor ablation [23,24,25]. Combined with surgery, radiation therapy, and chemotherapy, it can significantly improve the cure rate of malignant tumors and provide a novel therapeutic approach for treating tumors [26,27]. Furthermore, it was discovered that the oxygen enhancement ratio of RT was between 2.5 and 3.0, indicating that the sensitivity of normal oxygen cells was significantly greater than that of hypoxic cells [12,13]. In contrast, hyperthermia could increase the sensitivity of hypoxic cells to temperature, causing hypoxic tumor cells to be severely damaged. In addition, studies have demonstrated that hyperthermia can inhibit the cell cycle in G2/M phase cells that are sensitive to RT while acting on G1/S phase cells resistant to RT [28]. Consequently, combining local MW hyperthermia and RT can realize their complementarity, increase local blood flow, and solve hypoxic cell problems, thereby enhancing RT’s sensitivity.

Sorafenib (SFN) is an oral multitarget multikinase inhibitor that inhibits tumor growth, metastasis, and angiogenesis while inducing apoptosis [29,30]. SFN can inhibit Janus kinase (JAK), signal transducers, and activators of transcription (STAT) signaling pathways, resulting in hepatocellular death and inhibiting the proliferation of hepatocellular carcinoma [31,32]. It has been found [33] that SFN can promote apoptosis, inhibit angiogenesis, and inhibit mitogen-activated protein kinases in liver cancer [34]. Studies have also confirmed that SFN induces apoptosis in gastric cancer cells and dose-dependently inhibits cell proliferation [35,36,37]. In vitro and in vivo studies have demonstrated that SFN has antiproliferative and antiangiogenic effects on thyroid cancer cells [38]. Recent research has indicated that SFN can inhibit the crucial cysteine/glutamate reverse exchange system during glutathione (GSH) synthesis, thereby depleting GSH in tumor cells [39,40,41]. We conceived of using SFN to deplete intracellular GSH in order to achieve an efficient radiosensitization effect [39]. However, SFN’s therapeutic window is severely constrained by its high toxicity and low bioavailability. Poly (lactic-co-glycolic acid) (PLGA) is a biocompatible, biodegradable, high-polymer organic compound synthesized from lactic acid and glycolic acid that has found widespread application in pharmaceutical biology. SFN encapsulated in PLGA can effectively increase its bioavailability, and is anticipated to be an effective strategy for consuming glutathione in tumor cells in order to increase cell sensitivity to radiation and enhance the effect of RT.

**Scheme 1:**
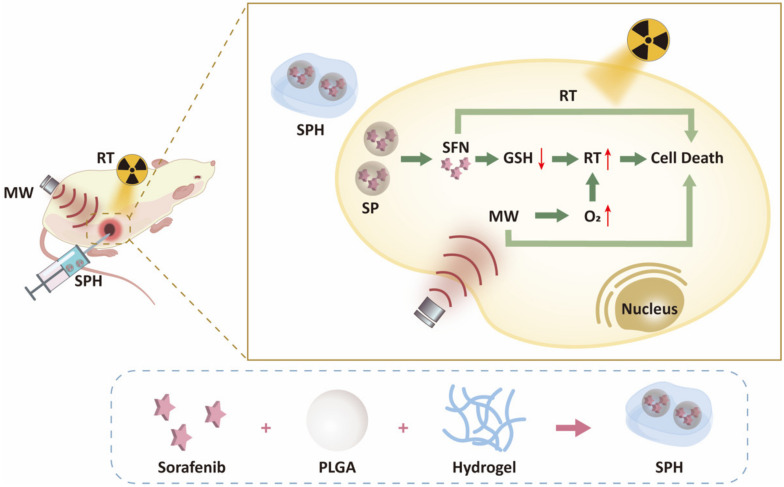
A schematic illustration of PLGA loading sorafenib hydrogel nanoplatform, combined with microwave hyperthermia for double radiotherapy sensitization.

For RT sensitization, we developed an SFN-loaded PLGA hydrogel system (SPH) in conjunction with MW hyperthermia (Scheme 1). A SPH with stable physiological properties was produced by combining SFN and PLGA in a specific proportion and encapsulating the mixture in a temperature-sensitive hydrogel. After intratumoral injection of SPH combined with MW hyperthermia in mice, SPH can not only directly cause thermal damage to tumor cells, but also increase blood oxygen delivery due to local heating of the tumor site, thereby overcoming the problem of intratumoral hypoxia and achieving “first” RT sensitization. In contrast, high temperatures can dissolve the hydrogel and release SFN. In addition to inhibiting tumor growth and cell growth, SFN can also achieve the “second” RT sensitization by inhibiting the synthesis of reduced GSH in cells and increasing ROS content. Additionally, the prepared hydrogel system can achieve a single dose for multiple treatments, significantly reducing patient discomfort. Experiments both in vitro and in vivo have demonstrated that SPH, combined with hyperthermia, can achieve a double RT sensitization effect and has a significant tumor-inhibiting effect when combined with RT. In conclusion, the SPH nanosystem combined with thermoradiotherapy is a promising course of combined tumor therapy developed by our team.

## 2. Materials and Methods

### 2.1. Reagents

Agarose was purchased from Yare Shanghai (Shanghai, China). PLGA and PLA were obtained from Chongqing Yusi medical technology cable Co., Ltd. (Chongqing, China). A GSH Assay Kit was purchased from Beyotime Institute of Biotechnology (Shanghai, China). 2′,7′-Dichlorofluorescin diacetate (DCFH-DA) was obtained from Solarbio life sciences (Beijing, China). The other reagents used in this work were purchased from Sinopharm Chemical Reagent (Shanghai, China) and Aladdin-Reagent (Shanghai, China).

### 2.2. Cell Culture

The 4T1 cancer cell line was obtained from the Cell Bank of the Chinese Academy of Sciences and incubated in RPMI-1640 medium supplemented with 10% FBS in a humidified atmosphere at 37 °C. Cell cultures under normoxic conditions (pO_2_: 21%) were maintained in a humidified incubator at 37 °C, with 5% CO_2_ and 95% air. Hypoxic conditions (pO_2_: 2%) were simulated by placing cells in a hypoxic incubator (Moriguchi, Japan) in a mixture of 2% O_2_, 5% CO_2_, and 93% N_2_.

### 2.3. Preparation and Characterization of Sorafenib-Loaded PLGA Nanoparticles (SP NPs) and Sorafenib-Loaded PLA Nanoparticles (SA NPs)

The PLGA NPs were prepared using an oil in water (O/W) emulsion method [42]. A total of 50 μL of SFN (5 mg/mL in DMSO) was added to 1 mL PLGA (5 mg/mL in dichloromethane). Then, the mixture was homogenized and the organic solvent evaporated. SP NPs were obtained after centrifugation at 3500× *g* for 20 min. SA NPs were prepared by the same method used for SP NPs, except that PLGA was replaced with PLA.

The morphology structures of SP NPs and SA NPs were detected by the TEM (JEOL-2100). Specifically, 10 μL of 100 μg/mL SP or SA PBS solution was sucked onto the carbon support membrane (Electron Microscopy China), and then dried for the TEM test. UV-vis spectra of samples were recorded by the UV-vis spectrophotometry Lambda 35 (Perkin-Elmer, Waltham, MA, USA). The diameter of the nanoparticles was measured by dynamic light scattering (DLS, Nano-Zen 3600, Malvern Instruments, Malvern Worcestershire, UK).

### 2.4. Drug Release Study

The release profiles of sorafenib-loaded PLGA NPs were investigated in PBS at 37 °C. Briefly, 1 mL SP NPs (0.1 mg) in PBS were put in a shaker incubator at 37 °C. At the designed time points, SP NPs were centrifuged at 5000 rpm for 20 min, then the supernatant was analyzed by a UV spectrophotometer at 275 nm to calculate the SFN release content. 

### 2.5. Preparation and Characterization of SPH

The general protocol for the hydrogel preparation was as follows. The prepared SP NPs or SA NPs (2 mg) were added into 10 mL 2% agarose solution to form SPH or SAH. Then, 5 mL SPH and SAH were freeze-dried, and a scanning electron microscopy (SEM) test was conducted. SEM images were captured on a Hitachi FE-SEM S4800 instrument with an acceleration voltage of 3 kV. Rheology experiments were performed on HAAKE MARS60 (Thermo Fisher Scientific, Waltham, MA, USA), according to our previous work [43].

### 2.6. Microwave Heating and Drug Release Study Experiment In Vitro

Next, the SPH (3 mL) was irradiated by MW at different powers (0.3 W, 0.6 W, 0.9 W, and 1.5 W) for 5 min, and the temperature rise was recorded in 30 s intervals by a forward-looking infrared (FLIR) imaging instrument. The temperature change curves of time were plotted to assess the MW thermal properties.

To study the drug release of SPH under microwave radiation, the drug release experiment was first conducted for 10 min without 0.9 W microwave radiation. After receiving 3 min of microwave radiation, the drug release experiment was conducted for 10 min without microwave radiation, and four cycles were carried out. At appropriate time points, different samples of 100 μL were collected for the shaking experiment, and the content of the released drug was monitored by a UV-visible spectrophotometer.

### 2.7. In Vitro Colony Formation Assay

The 4T1 cells growing during the logarithmic growth period were prepared into a single-cell suspension, which was counted, placed into a 6-well plate, and incubated at 37 °C with 5% CO_2_ for 24 h. After changing the medium, the cells were first incubated with 5 groups of media with different concentrations SFN (0, 50, 100, 200 µg/mL) of SPH under normal oxygen conditions: (1) PBS; (2) RT; (3) MW + RT; (4) SPH + RT; and (5) SPH + MW + RT. After 24 h of culture, the culture medium was transferred to the new medium and washed 3 times. The cells in groups 3 and 5 were incubated for 5 min under microwave radiation of 0.9 W, and then groups 2, 3, 4, and 5 were irradiated with X-rays at a dose of 4Gy. Then, the cells were washed with PBS, and the medium was changed every 3 days for 10 days. Finally, the cells were cloned using 4% paraformaldehyde and stained with Giemsa. Colonies with more than 50 cells were counted under the microscope, and the survival fractions (SF) were calculated using the formula SF = colonies counted/cells seeded. Then, the procedure was repeated under hypoxic conditions. To simulate the process of hypoxia, the petri dishes were sealed with liquid paraffin and oxygen was cut off from the cells.

### 2.8. In Vitro ROS Generation

ROS generation was also assessed in vitro on 4T1 cells. The intracellular generation of ROS was detected utilizing a DCFH detection kit. Afterwards, cells were incubated in 5 different groups: (1) PBS; (2) RT; (3) MW + RT; (4) SPH + RT; and (5) SPH + MW + RT. The concentration of SPH in groups 4 and 5 was 100 µg/mL. After 24 h of culture, the culture medium was transferred to the new medium and washed 3 times. The cells in groups 3 and 5 were incubated for 5 min under microwave radiation of 0.9 W, and then groups 2, 3, 4, and 5 were irradiated with X-rays at a dose of 4Gy. After 12 h of incubation, the DCFH-DA detection kit was added. Then, cells were detected under a fluorescent microscope (IX81, Olympus, Tokyo, Japan).

### 2.9. Detection of Intracellular GSH

The commercially available GSH assay kit was used to detect the depletion of GSH. 4T1 cells were incubated in 5 different groups at different concentrations of SFN (0, 50, 100, 200 µg/mL) and of SPH: (1) PBS; (2) RT; (3) MW + RT; (4) SPH + RT; and (5) SPH + MW + RT. After 24 h of culture, the culture medium was transferred to the new medium and washed 3 times. The cells in groups 3 and 5 were incubated for 5 min under microwave radiation with a power of 0.9 W, and then groups 2, 3, 4, and 5 were irradiated with X-rays at a dose of 4Gy. After 12 h of incubation, the GSH content was measured by employing a commercial colorimetric GSH assay kit. The assay was carried out according to the manufacturer’s instructions. An absorbance of 270 nm was measured by a microplate reader.

### 2.10. γ-H_2_AX Immunofluorescence Analysis In Vitro

4T1 cells were seeded in confocal dishes at a density of 1 × 10^6^ cells per dish, and then incubated at 37 °C with 5% CO_2_ for 24 h. After changing the medium, the cells were first incubated with 5 groups of media with SPH under normal oxygen conditions: (1) PBS; (2) RT; (3) MW + RT; (4) SPH + RT; and (5) SPH + MW + RT. The SFN concentration was 100 µg/mL. After 24 h of culture, the culture medium was transferred to the new medium and washed 3 times. The cells in groups 3 and 5 were incubated for 5 min under microwave radiation of 0.9 W, and then groups 2, 3, 4, and 5 were irradiated with X-rays at a dose of 4Gy. Then, γ-H_2_AX immunofluorescence analysis was conducted according to the previous work [44].

### 2.11. Animal Tumor Models

Female BALB/c mice aged 4 weeks were purchased from Vital River Company (Beijing, China). First, 100 μL of 4T1 cell suspension (1 × 10^6^ cells per mL) was subcutaneously injected into each mouse to establish the tumor models. The animal experiments were carried out according to the protocol approved by the Ministry of Health in the People’s Republic of PR China (document no. 55, 2001) and were approved by the Administrative Committee on Animal Research of the Shenzhen People’s Hospital.

### 2.12. In Vivo Infrared Thermography

To monitor the in vivo photothermal effect, SPH was intratumorally injected into the tumor-bearing mice, and the tumors were then subjected to 0.9 W microwave radiation for 5 min at 1 h post-injection. The rises in temperature were recorded at 30 s intervals by a forward-looking infrared (FLIR) imaging instrument. The PBS-injected mice were used as control group. 

### 2.13. In Vivo Antitumor Study

The mice were first divided randomly into 5 groups (each group included 5 mice): (1) PBS; (2) RT; (3) MW + RT; (4) SPH + RT; and (5) SPH + MW + RT. Groups 4 and 5 were injected with SPH (6 mg/kg SFN). After 12 h of injection, the mice in groups 3 and 5 were subjected to microwave radiation at a power level of 0.9 W for 5 min; then, groups 2, 3, 4, and 5 were irradiated with X-rays at a dose of 4Gy. The body weights and tumor volumes in the mice in all groups were monitored every 2 days. A caliper was employed to measure the tumor length and tumor width, and the tumor volume was calculated according to following formula: Tumor volume = tumor length × tumor width^2^/2. After 16 days of treatment, the mice were sacrificed. Five main organs (heart, liver, spleen, lung, and kidney) of all mice were harvested, washed with PBS, and fixed with paraformaldehyde for histology analysis. The tumor tissues were weighed and fixed in 4% neutral buffered formalin, processed routinely into paraffin, and sectioned at 4 μm. Then, the sections were stained with H&E and DHE, and were finally examined by using an optical microscope (BX51, Olympus, Tokyo, Japan) and a fluorescence microscope (IX81, Olympus, Tokyo, Japan).

### 2.14. In Vivo Tumor Vascular and Hypoxia Microenvironment Study

Healthy Balb/c mice were given intratumoral SPH (6 mg/kg SFN). At 12 h after injection, the mice were subjected to infrared thermal radiation with a power level of 0.9 W and X-ray radiation at a dose of 4Gy. The healthy Balb/c mice were given intratumoral PBS as control. HIF-1α immunofluorescence histochemical staining was conducted according to the standard protocol. The cryosections were observed by a confocal laser scanning microscope (CLSM; IX81, Olympus, Tokyo, Japan).

### 2.15. In Vivo Toxicity

Healthy Balb/c mice were given intratumoral SPH (6 mg/kg SFN, n = 3). At 12 h after injection, the mice were subjected to infrared thermal radiation with a power level of 0.9 W and X-ray radiation at a dose of 4Gy for 5 min every 3 days. Three healthy Balb/c mice were selected as controls. After 15 days of treatment, mouse blood samples were collected for blood biochemical analysis. The core, liver, spleen, lung, kidney, and other major organs were taken, fixed with 4% formalin, embedded in paraffin, sliced into 4 µm slices, stained with hematoxylin and eosin (H&E), and observed with an optical microscope (BX51, Olympus, Tokyo, Japan).

### 2.16. Statistical Analysis

Data analyses were conducted using the GraphPad Prism 5.0 software. The significance between each two groups was calculated by the Student’s *t*-test. * *p* < 0.05, ** *p* < 0.01, *** *p* < 0.005.

## 3. Results and Discussion

**Figure 1:**
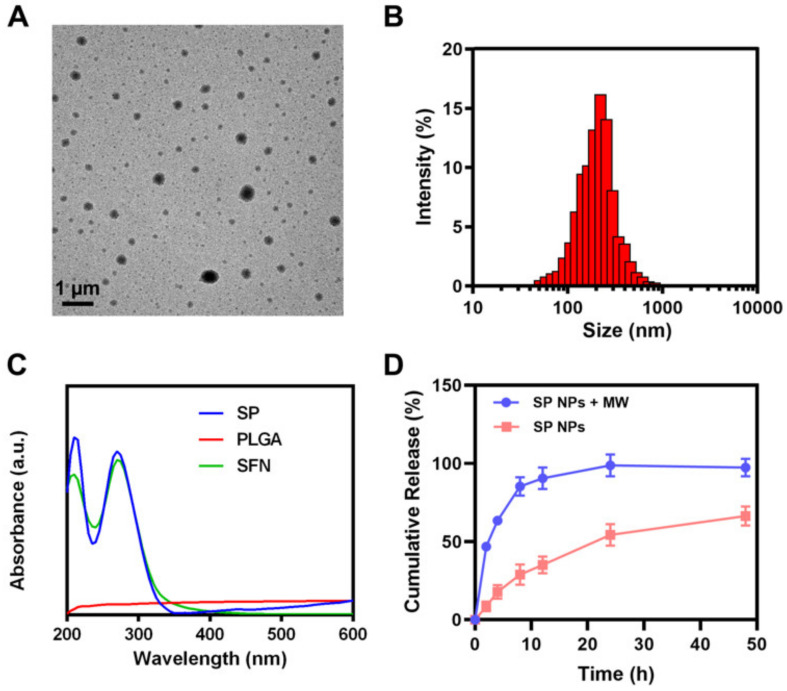
(**A**) TEM image of SP NPs. (**B**) Size distribution of SP NPs (containing 0.01 mg/mL SFN). (**C**) UV−vis spectrum of SP, PLGA, and SFN. (**D**) Cumulative SFN release from SP with or without MW (0.9 W).

According to the literature [45], SFN-loaded PLGA (SP) NPs were prepared for the following in vitro and in vivo studies. Using a transmission electron microscope, prepared SP NPs were examined (Figure 1A). The SP NPs had a mean diameter of 230.9 ± 7.6 and were spherical and well dispersed (Figure 1B). Figure 1C depicts the UV-visible absorption spectra of SP and SFN, which were analyzed to determine whether SFN was successfully loaded into PLGA. Similar characteristic peaks between the two indicate that SFN was successfully loaded. We then examined the drug-release properties of SP NPs. Figure 1D depicts the drug release curves of SP NPs under different conditions. Under MW radiation, the SP NPs SFN release rate was significantly greater than under normal conditions. For example, at 48 h, the cumulative release rates of SP NPs + MW and SP NPs were, respectively, 97.3% and 66.4%. This may be because an increase in temperature under MW makes PLGA more susceptible either to degradation or to the formation of pores favorable to drug release, resulting in a rapid increase in drug release rate. We further studied the formation of nanoparticles (SA NPs) by wrapping SFN with another kind of polymer micelle PLA, and then prepared the agarose-wrapped SA system (SAH) with the same method as that used for PLGA (as shown in Figures S1 and S2). The TEM test showed that SA had a smaller size than SP, but SFN released in SAH was similar to that in SPH, and was also shown to have temperature-sensitive characteristics (Figure S3).

**Figure 2:**
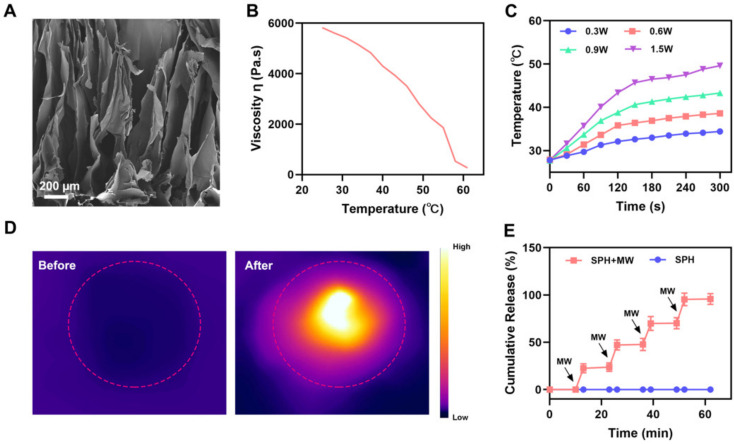
(**A**) Representative SEM images of SPH (containing 0.01 mg/mL SFN). (**B**) Viscosity measurements of reversibility for SPH during temperature jumps, from 25 °C to 61 °C. (**C**) Heating curves of SPH under varying powers of MW (containing 0.01 mg/mL SFN). (**D**) The infrared thermal images of the prepared SPH before and after MW treatment (MW power: 0.9 W). (**E**) The SFN release profile under different conditions; n = 3 (containing 0.01 mg/mL SFN).

The SPH system was created by combining agarose hydrogel with previously prepared SP NPs in a specific ratio and stirring at a constant rate of 60 degrees. The hydrogel preparation method is straightforward, rapid, biocompatible, and highly stable, with minimal side effects and low cytotoxicity. The in situ injection of hydrogel for tumor treatment can significantly reduce the drug dose and effectively prevent the toxic side effects caused by drug diffusion within the body [43,46,47]. The continuous enrichment and release of hydrogel-loaded drugs at the tumor site can accomplish the goal of multiple drug administration and effectively alleviate patients’ pain. Figure 2A illustrates the SEM images of the prepared SPH. Due to the temperature sensitivity of the agar-hydrogel, we tested the rheological properties of SPH at various temperatures, as shown in Figure 2B. As the temperature rises, the stored modulus value of SPH will gradually decrease, indicating that it will dissolve at high temperatures and release the contained SP NPs, as predicted. As previously mentioned, MW hyperthermia can convert MW energy into heat, causing the temperature of the tumor tissue to rise and remain elevated for some time, thereby inducing apoptosis in cancer cells. We investigated the temperature variation of the SPH system under varying MW radiation intensities. As shown in Figure 2C, when the MW power is increased from 0.3 to 1.5 W, the temperature of each group rises over time. After 5 min of radiation at 1.5 W, the temperature can reach nearly 50 °C, and the temperature rises to nearly 22 °C. When the radiation is 0.9 W, the temperature rises 15.6 °C within 5 min and can reach 43 °C at its maximum. According to certain studies, when the surrounding tissue of a tumor is heated to 42 °C, the tumor temperature can reach 43 to 48 °C. This temperature difference can kill cancer cells without harming healthy cells because hypoxic and acidic tumor cells are sensitive to heat, which results in a selective effect on tumor cells. Under the influence of high temperature, the blood vessels of the normal tissue surrounding the tumor dilate, the blood flow is accelerated, and the oxygen concentration at the tumor site is increased, allowing for adequate radiation sensitization. To further evaluate the MW thermal performance of SPH, infrared thermal imaging was performed on the prepared SPH for 5 min before and after MW radiation with a power of 0.9 W, as shown in Figure 2D. It indicates that the temperature of the prepared SPH system increased significantly with MW radiation and dissolved gradually. As shown in Figure 2E, we evaluated SPH’s ability to regulate drug release. Under MW radiation, SPH generates a thermal response, and the dissolution of the hydrogel accelerates the release of SFN. Upon cessation of MW radiation, the hydrogel cools and continues to store and protect the drug. This suggests that our SPH system can function as a thermal switch to reliably regulate drug release in a therapeutic setting.

**Figure 3:**
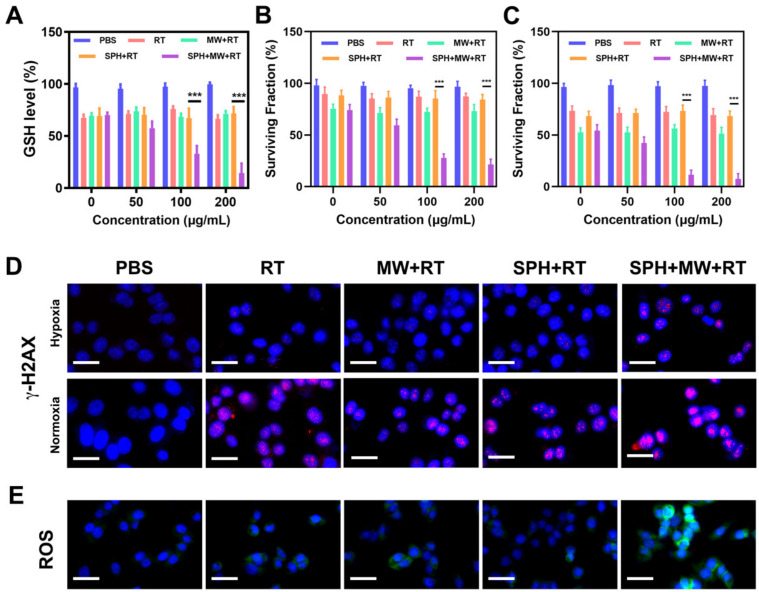
(**A**) The effect of different formulations on intracellular GSH levels (n = 3). (**B**) Colony formation assay results for 4T1 cells after different treatments under hypoxic conditions (n = 3). (**C**) Colony formation assay results for 4T1 cells after different treatments under normoxic conditions (n = 3). (**D**) γ-H_2_AX-stained 4T1 cells after different treatments under hypoxic or normoxic conditions. (**E**) Fluorescence images of ROS production in 4T1 cells following various treatments. (n = 3). Scale bars: 50 µm. * *p* < 0.05, ** *p* < 0.01, *** *p* < 0.005; Student’s *t*-test.

Considering that the SPH system is well-structured and performance-characterized, we investigated this system’s in vitro antitumor effects when combined with MW thermoradiation therapy. It is well known that high-level GSH within cancer cells can promote their survival and their resistance to radiation therapy by removing reactive oxygen species [48,49]. GSH consumption by tumor cells can effectively increase their sensitivity to RT [50,51]. Studies indicate that SFN can inhibit the cysteine (Cys) and glutamic acid (Glu) exchange and transport systems, thereby inhibiting GSH synthesis [40,41]. To examine the SPH system’s ability to deplete GSH, we measured the GSH levels of cells following various treatments: (1) PBS; (2) RT; (3) MW + RT; (4) SPH + RT; and (5) SPH + MW + RT. As shown in Figure 3A, neither the MW + RT nor the SPH + RT group appeared to significantly reduce GSH levels in cells compared to RT alone. Under MW thermal radiation, we hypothesized that the release of SFN from SPH could effectively inhibit GSH synthesis, as well as that the ROS produced by RT could effectively deplete GSH in tumor cells. After treatment with SPH + MW + RT, the intracellular GSH level decreased gradually. This indicates that SPH + MW + RT had an excellent capacity for GSH consumption. Then, the plate clone formation assays were used to evaluate the sensitization efficiency of each group under normoxic and hypoxic conditions (Figure 3B,C). Under hypoxic conditions, the control group’s cell viability remained unchanged. In addition, MW + RT inhibited more tumor growth than RT alone. The SPH + MW + RT group exhibited the most pronounced tumor growth inhibition effect, and the effect was concentration-dependent. The results showed that SPH + MW + RT had the best tumor growth inhibition rate. Since oxygen deficiency induces tumor cell resistance to RT, ameliorating oxygen deficiency can significantly increase the sensitivity of RT [44,52]. Under normoxic conditions, the inhibiting effect of SPH + MW + RT groups on tumor growth was significantly enhanced compared to under hypoxic conditions. Double-stranded DNA break (DSB) formation in tumor cells can indicate radiation sensitization [53]. As shown in Figure 3D, we detected the fluorescence of γ-H_2_AX in the nuclei of various groups under normoxic and hypoxic conditions. Under hypoxic conditions, RT encountered substantial RT resistance, and a negligible amount of DSB was produced. Compared to RT alone, MW + RT or SPH + RT did not lead to a significant increase in double-strand breaks (DSB). SPH + MW + RT resulted in the most pronounced DNA damage, indicating that SPH + MW + RT can effectively increase the sensitivity to the RT effect. Compared to hypoxic conditions, the quantity of DSB was significantly higher in SPH + MW + RT under normoxic conditions. This also confirms that improving hypoxia can, indeed, significantly improve the sensitivity of RT. We further compared the test results by determining whether each group produced ROS. As shown in Figure 3E, the absence of fluorescence production in the blank group indicates that ROS levels in cancer cells are negligible. The RT, MW + RT, and SPH groups did not significantly increase ROS production. The SPH + MW + RT group exhibited the strongest fluorescence signal, indicating a significant increase in ROS production. These results demonstrate the superior antitumor effect of our treatment regimen in vitro and encourage us to continue investigating its efficacy in vivo.

**Figure 4:**
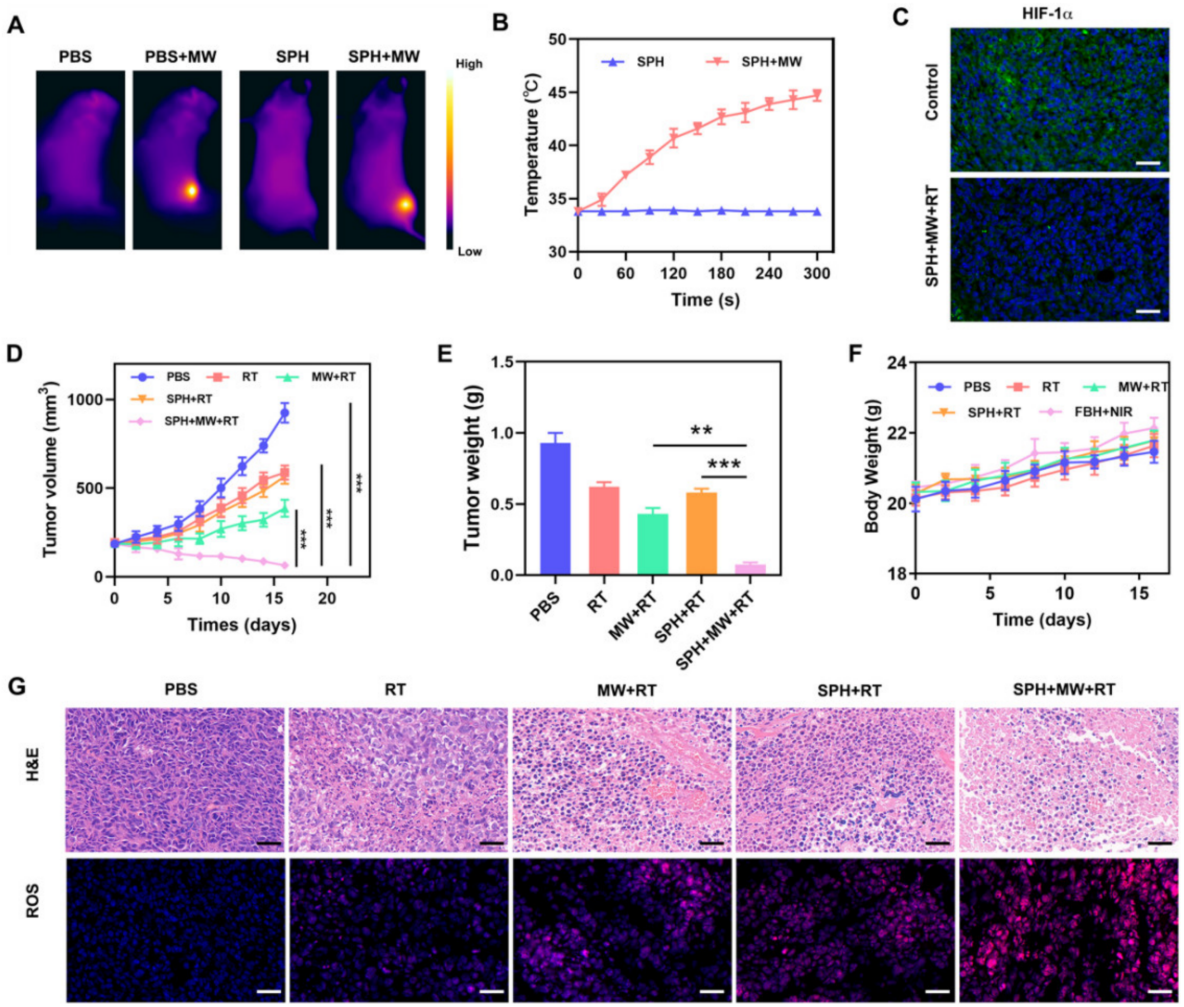
(**A**) Infrared thermal images of tumors in the specified treatment groups of 4T1 tumor-bearing mice before and after 5 min of MW (0.9 W) radiation (n = 5). (**B)** Temperature curves of 4T1 tumor-bearing mice in the specific treatment group within 5 min of MW (0.9 W) radiation (n = 5). (**C**) HIF-1 was immunofluorescence-detected in the indicated treatment groups (n = 5). (**D**) Tumor volume and (**E**) tumor weight following the indicated treatments (n = 5). (**F**) Body weight following the indicated treatments (n = 5). (**G**) HPF and H&E-stained tumor sections from the indicated treatment groups (n = 5). Scale bars: 100 µm. ** *p* < 0.01, *** *p* < 0.005; Student’s *t*-test.

**Figure 5:**
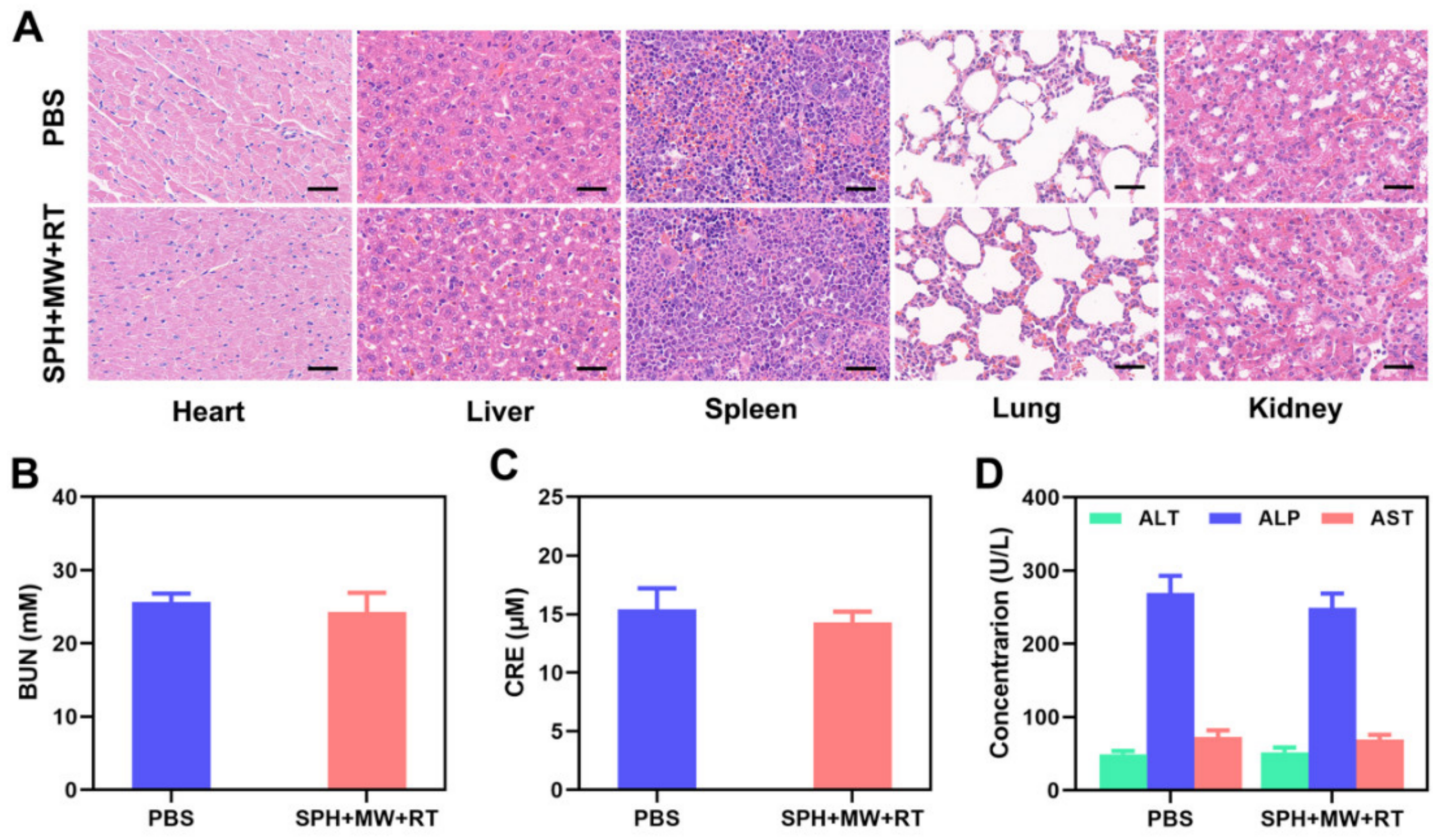
(**A**) H&E-stained images of the mice’s major organs, including the heart, lungs, liver, kidneys, and spleen, after exposure to various therapies 16 days post-injection (n = 5). (**B**) Kidney function markers: BUN, (**C**) CRE; (**D**) liver function indicators: ALT, AST, and ALP, following different treatments (n = 5). Scale bars: 100 μm.

We have demonstrated that MW has an excellent heating effect in vitro, so we first examined the heating effect of MW in vivo to determine whether it was selective for different reagents. Two groups of mice with 4T1 tumors were established. PBS and SPH were, respectively, injected into the tumor. At 24 h after injection, the two groups’ temperature changes at the tumor site were recorded every 30 s for 5 min using an infrared thermal imager, under MW radiation at 0.9 W. As shown in Figure 4A,B, the tumor temperature of mice injected with PBS or SPH significantly increased when exposed to MW radiation. Within 5 min, the tumor temperature in the SPH group increased from 33.8 to 44.7 °C, and the tumor temperature increased significantly even in mice injected with PBS. In contrast, the tumor temperature in mice not exposed to MW radiation was negligible. This increase can, on the one hand, cause the temperature-sensitive hydrogel to dissolve and release the SFN for the following action. On the other hand, it can increase the blood flow in the tumor site, thereby increasing the oxygen enrichment degree and resolving the problem of oxygen deficiency at the tumor site. We examined the effects of MW hyperthermia on the upregulation of hypoxia-inducing factor (HIF-1) and RT hypoxia resistance in the tumor microenvironment. As shown in Figure 4C, the expression of HIF-1a was significantly downregulated in the MW treatment group compared to the control group, indicating that MW radiation could effectively combat the hypoxic tumor microenvironment, thereby achieving the sensitization effect of RT. Considering that MW hyperthermia can effectively resolve the problem of RT resistance caused by tumor hypoxia, we investigated the in vivo tumor activity of SPH and MW combined with RT. Mice with 4T1 tumors were treated with PBS, RT alone, RT combined with MW radiation, RT combined with SPH, and SPH combined with RT and MW radiation for this purpose. The tumor volumes were then measured 16 days later in each treatment group to evaluate the effect of the treatment (Figure 4D). Compared to the rapid tumor growth in the PBS group, the growth of the RT tumor alone was marginally slower, but not enough to influence subsequent tumor growth. However, the combined effect of RT and SCH was marginally superior to that of RT alone. This was due to the slow release of SFN from SPH by biological enzymes in vivo, which consumed a small amount of GSH at the tumor site, thereby increasing sensitivity to RT. Under the combined treatment of MW radiation and RT, tumor growth was inhibited to a certain degree, which was consistent with the ability of MW hyperthermia to solve the problem of hypoxia at the tumor site and thereby sensitize RT. After injection of SPH, tumor growth in mice was significantly inhibited after combined treatment with MW radiation and RT. SPH was able to achieve a double RT sensitization effect in an MW hyperthermia environment, demonstrating that combined RT can ablate tumors effectively, as is consistent with tumor weight measurements on mice weighed after treatment (Figure 4E). During the study, none of these treatments resulted in significant changes in the body weight of the mice (Figure 4F), indicating the absence of significant systemic toxicity. In addition, ROS production was most pronounced in the SPH + MW + RT treatment group compared to other treatments, demonstrating that our treatment had the greatest effect (Figure 4G). Hematoxylin and eosin (H&E) staining of tumor tissues demonstrated that the SPH + MW + RT group exhibited the highest levels of tumor necrosis and apoptosis, demonstrating that SPH + MW can significantly augment the effect of RT. In conclusion, these results demonstrate the benefits of our SPH effect, which integrates MW and RT, and confirm that the combination of SPH + MW and low-dose RT can significantly inhibit the growth of tumor tissue. Last but not least, we collected the major organs of these mice and discovered no apparent inflammatory damage or histological changes, indicating that our experimental protocol was not toxic (Figure 5A). In addition, hematological and biochemical analyses (Figure 5B–D) demonstrated that the regimen exhibited no hepatotoxicity or nephrotoxicity of significance. These results suggest that our proposed SPH-mediated combined MW and RT method is biosafe and inhibits tumor growth, making it a promising anticancer treatment.

Our method focuses on the regulation of the tumor microenvironment, especially the treatment of tumors with high expression of GSH and hypoxia. Compared with the radiosensitizing effect of gold nanoparticles, our method has the advantages of better biosafety and tumor microenvironment regulation ability [54,55,56]. This method may further inhibit tumor recurrence and metastasis, and enhance tumor immunity. PLGA and sorafenib are approved by the FDA, and agarose can be absorbed and utilized by the body, so they have better clinical application potential than gold nanomaterials. 

## 4. Conclusions

In conclusion, we designed an SFN-loaded PLGA hydrogel system combined with MW thermal therapy to improve RT. Under MW radiation, the tumor area heated up locally, and the blood flow accelerated to deliver more oxygen to the tumor area. This method can effectively overcome the RT resistance caused by an anoxic tumor environment. At the same time, under the influence of high temperature, the SPH injected into the tumor can dissolve rapidly. The released SFN can exhaust the GSH at the tumor site, resulting in double RT sensitization. The SPH and MW thermodynamic RT combination demonstrated significant antitumor effects both in vivo and in vitro, with no discernible toxic side effects. This research introduces a novel concept for the clinical application of MW thermal therapy and RT sensitization, and we will continue to develop new RT sensitization methods in the future.

## Data Availability

The data presented in this study are available on request from the corresponding author.

## Supplementary Materials

The following supporting information can be downloaded at: https://www.mdpi.com/article/10.3390/pharmaceutics15020487/s1, Figure S1: TEM image of SA NPs; Figure S2: Representative SEM images of SAH. (Containing 0.01 mg/mL SFN); Figure S3: The SFN release profile from SAH under different conditions. *n* = 3. (Containing 0.01 mg/mL SFN).
