# Supplementary material for: PLGA Nanoparticles Loaded with Sorafenib Combined with Thermosensitive Hydrogel System and Microwave Hyperthermia for Multiple Sensitized Radiotherapy
Source: Pharmaceutics. 2023 Feb 1;15(2):487. doi: 10.3390/pharmaceutics15020487 (PMC9965455; doi:10.3390/pharmaceutics15020487)
Supplement: Supplementary file 1 [file pharmaceutics-15-00487-s001.zip › pharmaceutics-2119919-supplementary.pdf]

PLGA Nanoparticles Loaded with Sorafenib Combined with Thermosensitive Hydrogel System and Microwave Hyperthermia for Multiple Sensitized Radiotherapy

Ziqi Wang [1,†], Bo Liu [1,†], Jingyao Tu [1,†], Jingfeng Xiang [2], Hui Xiong [3], Yue Wu [4], Shuaijie Ding [5], Daoming Zhu [2], Dongyong Zhu [6], Fei Liu [1,*], Guangyuan Hu [1,*] and Xianglin Yuan [1,*]

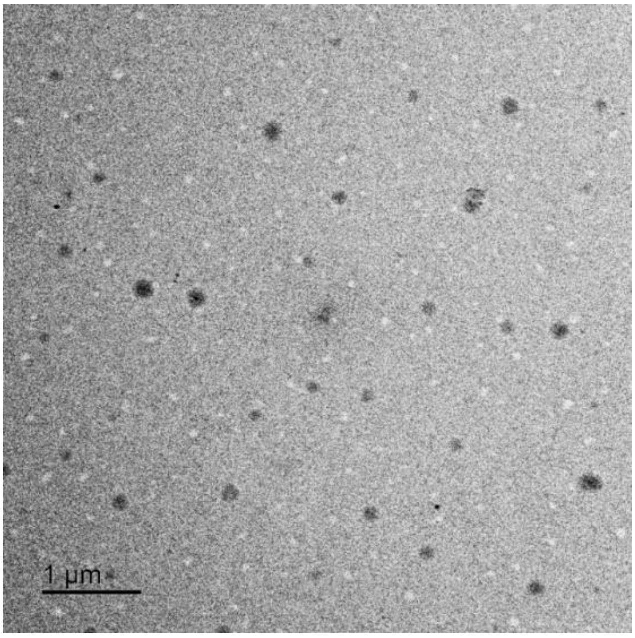

Figure S1. TEM image of SA NPs.

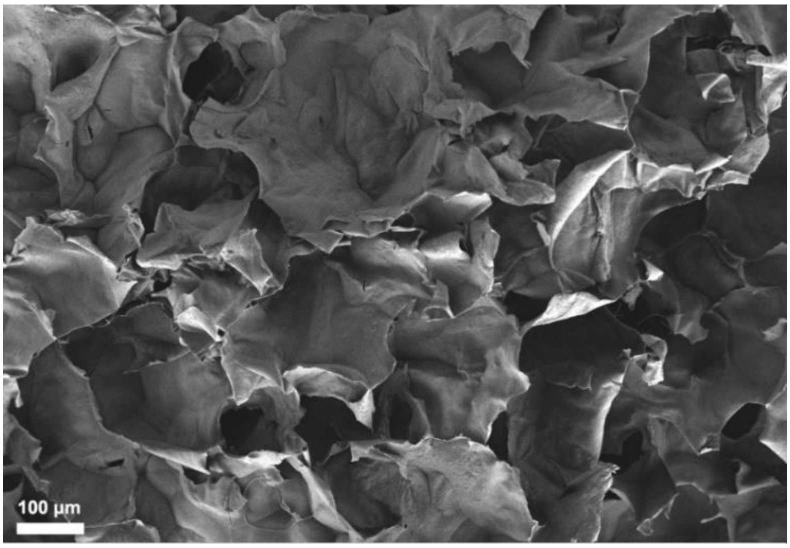

Figure S2. Representative SEM images of SAH. (Containing 0.01 mg/mL SFN).

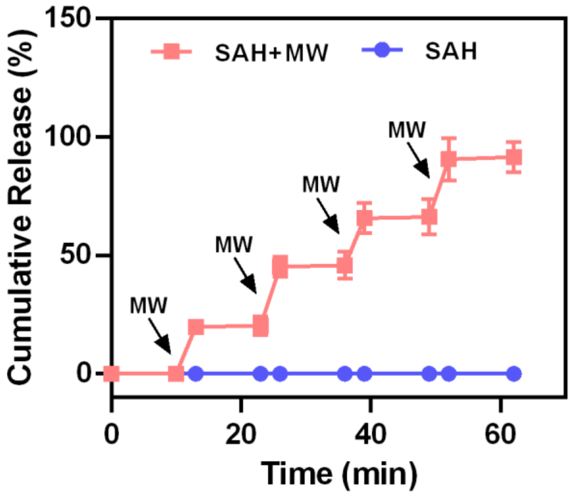

Figure S3. The SFN release profile from SAH under different conditions. n = 3. (Containing 0.01 mg/mL SFN).
